# Intra-specific variation in wing morphology and its impact on take-off performance in blue tits (*Cyanistes caeruleus*) during escape flights

**DOI:** 10.1242/jeb.126888

**Published:** 2016-05-01

**Authors:** Laura McFarlane, John D. Altringham, Graham N. Askew

**Affiliations:** Faculty of Biological Sciences, University of Leeds, Leeds LS2 9JT, UK

**Keywords:** Wing loading, Aspect ratio, Power, Aerodynamics, Kinematics

## Abstract

Diurnal and seasonal increases in body mass and seasonal reductions in wing area may compromise a bird's ability to escape, as less of the power available from the flight muscles can be used to accelerate and elevate the animal's centre of mass. Here, we investigated the effects of intra-specific variation in wing morphology on escape take-off performance in blue tits (*Cyanistes caeruleus*). Flights were recorded using synchronised high-speed video cameras and take-off performance was quantified as the sum of the rates of change of the kinetic and potential energies of the centre of mass. Individuals with a lower wing loading, WL (WL=body weight/wing area) had higher escape take-off performance, consistent with the increase in lift production expected from relatively larger wings. Unexpectedly, it was found that the total power available from the flight muscles (estimated using an aerodynamic analysis) was inversely related to WL. This could simply be because birds with a higher WL have relatively smaller flight muscles. Alternatively or additionally, variation in the aerodynamic load on the wing resulting from differences in wing morphology will affect the mechanical performance of the flight muscles via effects on the muscle's length trajectory. Consistent with this hypothesis is the observation that wing beat frequency and relative downstroke duration increase with decreasing WL; both are factors that are expected to increase muscle power output. Understanding how wing morphology influences take-off performance gives insight into the potential risks associated with feather loss and seasonal and diurnal fluctuations in body mass.

## Abstract (precis)

**Summary:** Blue tits with relatively larger wings have higher escape take-off performance, indicating that moult could increase the risk of predation.

## INTRODUCTION

Take-off is the means by which animals initiate flight and become airborne (Earls, 2000; Pennycuick, 2008). In some instances, take-off is also an important component of predator avoidance, and performing rapid take-off flights can increase an individual's chances of survival (Fernandez and Lank, 2007; Kullberg et al., 1998; Williams and Swaddle, 2003; Witter et al., 1994). Take-off performance is ultimately limited by the mechanical power available from the flight muscles, but how much power can be diverted to accelerating and elevating the centre of mass of the body (CoM) depends on how much power is required to impart momentum to the air and to overcome the drag on the wings and body (Norberg, 1990). The primary constraint during slow and take-off flight is aerodynamic lift generation to overcome the induced power requirements (Chai et al., 1999; Rayner and Swaddle, 2000). Lift must balance body weight and any acceleration force and is proportional to wing area and velocity^2^ (Norberg, 1990; Askew et al., 2001). As take-off is from a standing start and the forward velocity of the bird is low, the airflow deflected downwards by the wings (the induced velocity; Pennycuick, 2008) is determined largely by the flapping velocity and is therefore relatively low. Thus, wing morphology in relation to body mass becomes a critical factor in generating sufficient lift and has a major impact on the power required to fly and on flight performance. Avian wing morphology can be characterised in terms of two variables: wing loading (WL; body weight relative to wing area) and aspect ratio (AR; wing span^2^ relative to wing area). Having a low wing loading should therefore facilitate take-off performance by reducing the power needed to generate the induced velocity, allowing more of the power available to be used to accelerate and raise the CoM. Wings with a high aspect ratio may also facilitate take-off performance by reducing the induced power requirements. However, long wings may restrict wing stroke amplitude during take-off from the ground or increase the power required to accelerate the wings as a result of increased wing inertia.

Body mass varies as a result of both diurnal and seasonal deposition of fat, and wing area varies through the loss of feathers (and consequently wing area) as a result of moult and feather wear. Therefore, there is considerable intra-specific variation in wing morphology in relation to body mass. Wing loading increases by 60% in blackcaps prior to migration as a result of increased body mass (Kullberg et al., 1996) and by 10–25% in starlings and hummingbirds because of reduced wing area during moult (Rayner and Swaddle, 2000; Chai et al., 1999). It has been argued that birds must balance the benefits of maintaining low body mass to facilitate escape from predators with the reduced risk of starvation that results from increased fuel supplies. The increase in body mass that occurs in the absence of predators (Gosler et al., 1995) and the decrease in body mass in response to an increase in the perceived risk of predation (Gentle and Gosler, 2001; Zimmer et al., 2010, 2011) support this hypothesis. However, empirical data supporting a link between flight performance and WL is equivocal. Several studies report that flight performance is reduced in birds with a higher WL resulting from diurnal changes in body mass or wing area (simulated moult), consistent with the expected changes in performance (Kullberg et al., 1996; Lind et al., 1999; Macleod, 2006; Swaddle et al., 1999). However, other studies report no significant change in flight performance in relation to diurnal changes in body mass and wing loading (van der Veen and Lindstrom, 2000; Macleod, 2005, 2006; Williams and Swaddle, 2003). Not all studies have performed a complete biomechanical analysis of performance (e.g. some studies consider single components of performance such as flight velocity or take-off angle) and in some cases positional data have low time resolution. These factors may have obscured the relationship between WL and take-off performance. To our knowledge there has been no quantification of intra-specific variation in AR in relation to take-off performance.

List of symbols and abbreviations

*a*: acceleration of the centre of mass of the body
AR: aspect ratio
*b*: total wing span
*C*_D,par_: parasite drag coefficient of the body
*C*_D,pro_: profile drag coefficient of the wing
CoM: centre of mass of the body
*E*_K,ext_: kinetic energy of the centre of mass of the body
*E*_P_: potential energy of the centre of mass of the body
***g***: gravitational acceleration
*k*: induced velocity correction factor
*M*_b_: body mass
*n*: wing beat frequency
*P*_aero_: total aerodynamic power
*P*_CoM_: take-off power to change the potential and kinetic energy of the centre of mass of the body
*P*_ind_: induced power
*P*′_ind_: induced power required to create the induced velocity
*P*_par_: parasite power
*P*_pro_: profile power
*S*: total wing area
*t*: time
*t*_D_: downstroke duration
*t*_U_: upstroke duration
*T*: torque
*v*: velocity of the centre of mass of the body
*v*_min_, *v*_max_: minimum and maximum velocity of the centre of mass of the body
WL: wing loading
*x*, *y*, *z*: coordinates describing the three-dimensional position of the bird
*ẋ*, *ẏ*, *ż*: velocity of the centre of mass of the body in the *x*, *y* and *z* directions, respectively
β: stroke plane angle
θ: angle of elevation
τ: relative downstroke duration
Φ: wing beat amplitude
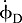
angular velocity of the wing during the downstroke (φ/*t*_D_)
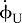
angular velocity of the wing during the upstroke (φ/*t*_U_)

The aim of our study was, therefore, to use a detailed kinematic analysis to determine the effects of intra-specific variation in wing morphology on take-off performance in wild-caught blue tits (*Cyanistes caeruleus*, Linnaeus 1758). By tracking the birds' flight trajectory using high-speed video recordings, take-off performance could be quantified as the rates of change of the kinetic and potential energies of the CoM of the bird during escape take-off flights. A full aerodynamic analysis was also performed in order to determine the total flight power requirements and therefore calculate the proportion of the total flight muscle power output used to accelerate and gain height. It was hypothesised that individuals with low WL and high AR would have higher take-off performance as a result of them being able to use a larger proportion of the power available from their flight muscles to accelerate and gain height, as a result of the relatively greater lift production by their wings.

## MATERIALS AND METHODS

### Animals and morphological measurements

Blue tits (*C. caeruleus*) were caught using mist nets, under licence from the British Trust of Ornithology (licence A issued to Chris Wright, University of Leeds) at two sites in North Yorkshire, UK (54°N 2°W Malham and 54°N 1°W Thorganby) between June 2011 and June 2013, during the months of June, July and September; one individual was caught in March. Individuals were sexed, aged (following Svensson, 1992; Jenni and Winkler, 1994) and weighed. A calibrated, digital photograph (Canon EOS 30D, Uxbridge, Middlesex, UK) was taken of the outstretched wing and body to calculate wing area (*S*) and span (*b*) (ImageJ, US National Institutes of Health, Bethesda, MD, USA; following Pennycuick, 2008). To confirm that differences in wing area were not due to variation in the wing position when being photographed or errors in the analysis of the wing photographs, images of re-captured birds were used to calculate wing area and determine the percentage difference in wing area determined from the two images. For each bird, wing loading (WL) and aspect ratio (AR) were calculated as (Norberg, 1990; Pennycuick, 2008):
(1)
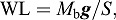

(2)
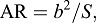


where *M*_b_ is body mass, ***g*** is gravitational acceleration, and *S* and *b* included both wings and the root box area (Pennycuick, 2008).

### Take-off flights and filming arena

Birds were placed inside a custom-built release box (internal dimensions: 17×19×12 cm, length×width×height) positioned 0.5–0.75 m off the ground. Once the bird had oriented towards the front of the box (the bird could be observed through a small Perspex window), the front and top of the box were triggered to spring open, releasing the bird and acting as a startle stimulus in an effort to elicit an alarmed and maximal take-off flight. Take-off flights were recorded using two, synchronised high-speed video cameras (Troubleshooter, Fastec Imaging, San Diego, CA, USA) operating at 250 frames s^−1^, shuttered at 0.4–0.8 ms (depending on light levels). The flight volume was calibrated via direct linear transformation using Matlab software (Matlab version R2009b, The MathWorks Inc., Natick, MA, USA) (Hedrick, 2008).

### Take-off analysis

Flights were analysed in cases where birds took-off instantly on opening of the release box and did not collide with the sides of the box. From the two synchronised video images, the estimated positions of the CoM (taken as the centre of an ellipsoid that encompassed the body), wing roots and wing tips of each bird were tracked, allowing the three-dimensional position of each digitised point to be calculated (Hedrick, 2008). In order to be able to attribute differences in take-off performance to differences in the aerodynamic forces produced by the wings, flights were digitised only once the feet had left the ground (Askew et al., 2001).

The coordinates of the CoM of the bird were plotted with respect to time for each flight and the velocities calculated by differentiating position in each axis dimension with respect to time. Individuals were compared by determining the velocities at an absolute distance of 0.5 m from the point of take-off. The overall velocity (*v*) of the bird was calculated from the velocities in the *x*, *y* and *z* directions as follows:
(3)
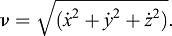


Take-off performance was quantified by calculating the rates of change of the potential energy (*Ė*_P_) and kinetic energy (*Ė*_K,ext_) of the CoM (Askew et al., 2001):
(4)
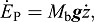

(5)
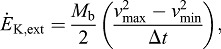


where *v*_max_ and *v*_min_ are the maximum and minimum velocity, respectively, and Δ*t* is the flight duration. In flights in which the bird decelerated, the rate of change of kinetic energy was defined as being negative. The total power of the centre of mass of the body (*P*_CoM_) was calculated as the sum of the rate of change of potential and kinetic energy (Eqns 4 and 5):
(6)
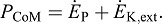


### Aerodynamic power

Only part of the power generated by the flight muscles can be used to accelerate and elevate the CoM; power is also required to overcome the drag on the wings and body and to generate the induced velocity. In order to understand differences in take-off performance, it may be informative to determine how the total aerodynamic power (a proxy measure of flight muscle power) is partitioned between different aerodynamic components. The aerodynamic power was calculated from the sum of the induced (*P*_ind_), profile (*P*_pro_) and parasite power (*P*_par_) (following Morris and Askew, 2010, where the equations are given in full). As the kinetic energy needed to accelerate the wings can potentially be recovered during late downstroke, the inertial power requirement was not calculated (Askew et al., 2001). In calculating aerodynamic power (*P*_aero_), an induced power factor of 1.2, a profile drag coefficient (*C*_D,pro_) of 0.02 (Rayner, 1979), body frontal area calculated following Pennycuick et al. (1988; see their eqn 6), and a parasite drag coefficient (*C*_D,par_) of 0.13 (Rayner, 1999) were assumed.

The analysis of take-off performance and calculation of the aerodynamic power components was carried out using Mathcad 15 (PTC, Needham, MA, USA).

### Statistical analysis

Statistical tests were conducted in Minitab 16 (Minitab Inc., State College, PA, USA). Data were tested for normality prior to statistical analyses. Parametric tests were used on normally distributed data; if not normally distributed, the data were transformed so as to normalise. In some instances, logarithmic, arcsine or square-root transformation still did not normalise the data and therefore non-parametric tests were used. The effects of month, time of day and site on body mass and wing morphology were determined by general linear model (GLM), with month and time of day included as random factors. As only one individual was caught in March, only the months of June, July and September were included and terms that did not improve the fit of the model were also removed from the analysis. Bonferroni or Tukey *post hoc* tests were used when the GLM returned a significant result. One-way ANOVA or Mann–Whitney *U*-tests were used to determine whether there were differences in the response and explanatory variables due to age or sex. There were no significant differences between adults and juveniles but there were some differences between males and females. A one-way ANOVA followed by a Tukey *post hoc* test was conducted to determine the differences in the power components. The least-squares regression slopes showing the relationships between *P*_aero_, *P*_CoM_ and the explanatory variables for males and females were also determined, as was the significance of the difference between the slopes. The test statistic was calculated as described by Zaiontz (http://www.real-statistics.com/regression/hypothesis-testing-significance-regression-line-slope/comparing-slopes-two-independent-samples/). As the slopes did not differ significantly, the relationships between *P*_aero_, *P*_CoM_ and WL, AR and the different wing beat kinematic variables were determined by GLM, with both season and site where the birds were collected included in the analysis on the pooled data. This meant that individuals that had been excluded because of indeterminate sex (sex can be difficult to determine outside the breeding season) could be included. All data are presented as means±s.e.m.

## RESULTS

### Intraspecific variation in morphology

**Fig. 1.**
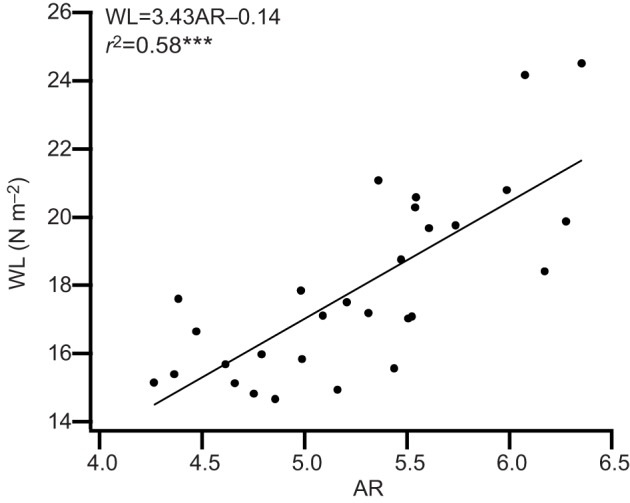
**Scatter plot showing the relationship between wing loading (WL) and aspect ratio (AR) in blue tits.** The solid line is a least-squares regression (***P<0.001, *N*=29).

A summary of the morphological characteristics of the population of birds used in this study is given in Table 1. Variation in wing area due to measurement error was assessed by comparing the areas obtained from re-captured birds (mean difference in wing area 1.9±0.94%, *N*=4). WL varied 1.7-fold across the individuals studied, ranging from 14.6 to 24.5 N m^−2^. Variation in WL occurred primarily as a result of differences in wing area and were related to the month (*F*_2,26_=13.15, *r*^2^=0.50, *P*<0.001) during which the bird was caught. Individuals with larger wing areas had lower WL (*F*_1,27_=137.39, *r*^2^=0.84, *P*<0.001), whereas *M*_b_ did not significantly affect WL (*F*_1,27_=0.13, *r*^2^=0.004, *P*=0.72). During June and September, birds had lower WL (approximately 17% lower) than during July (*P*<0.05). The inclusion of site, Malham or Thorganby, and time of day did not improve the GLM and they were therefore removed prior to the analysis. AR varied 1.5-fold across the individuals studied, ranging from 4.3 to 6.4, with the month during which the bird was flown having a significant effect (*F*_2,26_=8.02, *r*^2^=0.38, *P*<0.01), after removing site and time of day as neither improved the GLM. Differences in AR were due to variation in wing area (*F*_1,27_=12.27, *r*^2^=0.31, *P*<0.01) rather than wing span (*F*_1,27_=0.03, *r*^2^=0.01, *P*=0.87), with AR inversely related to wing area. Individuals had higher AR (*P*<0.05) during July compared with September (by approximately 14%), whereas birds flown during June had an intermediate AR, approximately 4% lower than in July but approximately 10% higher than in September. The *M*_b_ of an individual was not affected by the month (*F*_2,17_=0.15, *P*=0.86), time of day (*F*_8,17_=0.97, *P*=0.49) or the site (*F*_1,17_=0.13, *P*=0.76) at which a bird was captured. The differences in wing morphology and body mass for blue tits of different ages, sexes, sampling sites and times of the year are shown in Table 1. Blue tits with lower AR also had significantly lower WL (*F*_1,27_=37.33, *r*^2^=0.58, *P*<0.001; Fig. 1).

**Table 1.**
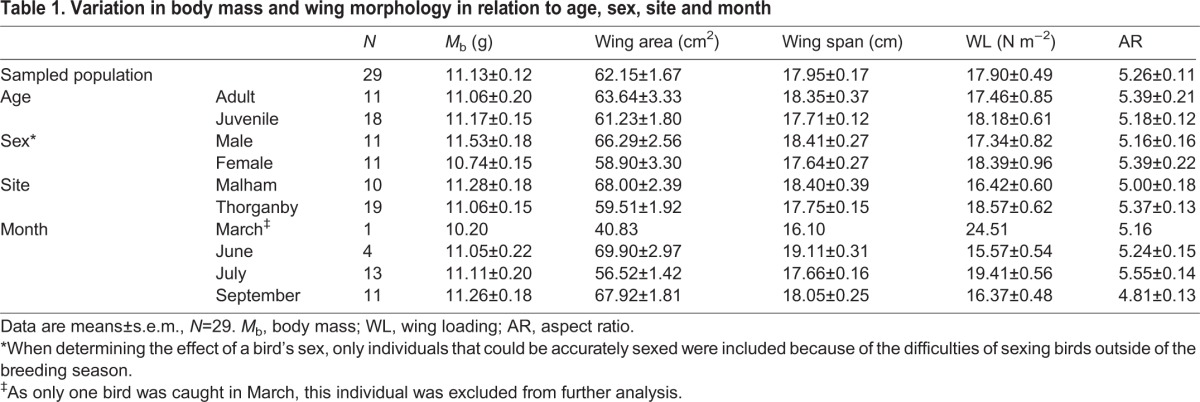
**Variation in body mass and wing morphology in relation to age, sex, site and month**

### Aerodynamic power components and overall flight performance

**Fig. 2.**
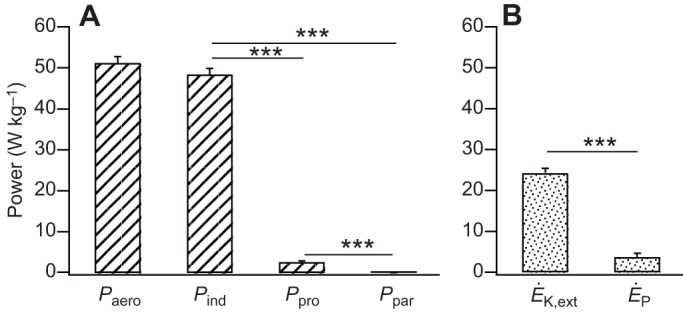
**Partitioning of the aerodynamic power requirements during escape take-off flight in blue tits.** (A) Total aerodynamic power (*P*_aero_) during take-off and its partitioning into separate aerodynamic components (induced power, *P*_ind_; profile power, *P*_pro_; parasite power, *P*_par_). (B) The apportioning of the total power of the centre of mass (*P*_CoM_) into the rates of change of potential and kinetic energies (*Ė*_P_ and *Ė*_K,ext_). Note that the power of the centre of mass is a component of the induced power (see Askew et al., 2001). Bars represent mean values with s.e.m., and significant differences are shown (***P<0.001, *N*=29).

The total aerodynamic power requirement of escape take-off flights in blue tits was 51.28±1.51 W kg^−1^ *M*_b_ (Fig. 2A). The total aerodynamic power was dominated by *P*_ind_ with relatively small amounts of power being needed to overcome the drag on the wings and body (*P*_ind_ was 95%, *P*_pro_ was 5% and *P*_par_ was 0.2% of *P*_aero_; Fig. 2A). This was expected given that during take-off flight from standstill, the forward motion of the bird is relatively slow and contributes a relatively small amount of circulation around the wings. As the *P*_pro_ and *P*_par_ requirements are a small proportion of *P*_aero_, no analysis of intraspecific variation in these power components was performed. The *P*_ind_ is the sum of the power required to increase the rate of change of potential (*Ė*_P_) and kinetic (*Ė*_K,ext_) energies of the CoM (*P*_CoM_) and that needed to generate the induced velocity (*P*′_ind_). Of the total *P*_aero_, approximately 55% (range 51–60%) was used to accelerate and increase the height of the CoM during take-off and approximately 39% (range 18–55%) was used to generate the induced velocity (*P*′_ind_; Askew et al., 2001). Of the power imparted to the CoM, 87% was the power associated with increasing the birds' kinetic energy and 13% was that associated with increasing the birds' potential energy (*t*_53_=14.20, *P*<0.001; Fig. 2B). Overall take-off velocity 0.5 m after take-off was 3.4±0.04 m s^−1^, acceleration was 10.3±0.3 m s^−2^ and the flight path had an average angle of elevation (θ) of 7.2±1.2 deg.

### Intra-specific variation in take-off performance, total aerodynamic power and wing beat kinematics

Males were heavier (*W*=158.5, *n*_1_=11, *n*_2_=11, *P*<0.05), and had a greater downstroke ratio (τ; *F*_1,21_=5.79, *P*<0.05) and higher *P*_aero_ (*W*=163.0, *n*_1_=11, *n*_2_=11, *P*<0.05) and *P*_CoM_ (*F*_1,21_=6.11, *P*<0.05) than females. However, when analysing the relationships between *P*_aero_ and *P*_CoM_, and *M*_b_, wing morphology and wing beat kinematics, the slopes for males and females did not differ significantly (Table S1). Therefore, all further analyses were carried out on the pooled blue tit data. Models testing for the relationship between *P*_aero_ (*F*_2,26_=0.28, *P*=0.76), *P*_CoM_ (*F*_2,26_=1.80, *P*=0.19) and WL, and between *P*_aero_ (*F*_2,26_=0.72, *P*=0.50), *P*_CoM_ (*F*_2,26_=0.62, *P*=0.55) and AR that included season and site did not improve the model's fit to the data and therefore season and site were removed.

**Fig. 3.**
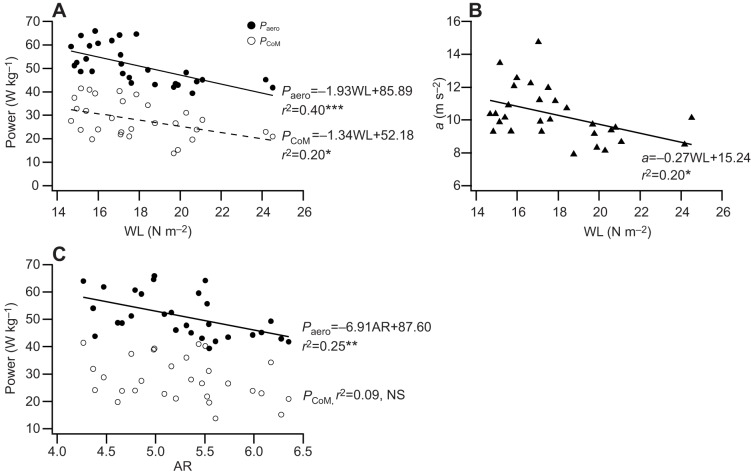
**The effects of wing morphology on take-off flight performance in blue tits.** Relationship between WL and (A) *P*_aero_ and *P*_CoM_, and (B) acceleration (*a*) of the CoM. Relationship between AR and (C) *P*_aero_ and *P*_CoM_. The solid lines are least-squares regressions (*P<0.05, **P<0.01 and ***P<0.001, *N*=29).

Intra-specific variation in wing morphology affected take-off performance. *P*_CoM_ increased significantly (*F*_1,27_=6.91, *r*^2^=0.20, *P*<0.05) with decreasing WL (Fig. 3A). Birds with the highest WL exhibited the lowest acceleration of the CoM (and consequently the lowest rate of change of *E*_K,ext_; *F*_1,27_=6.92, *r*^2^=0.20, *P*<0.05; Fig. 3B). Individuals with low WL had large wing areas and higher *P*_CoM_ (*F*_1,27_=7.24, *r*^2^=0.21, *P*<0.05); birds with a larger wing area also had a greater *P*_CoM_ (*F*_1,27_=6.16, *r*^2^=0.25, *P*<0.05). *P*_aero_ requirements during take-off increased with decreasing WL (*F*_1,7_=18.17, *r*^2^=0.40, *P*<0.001; Fig. 3A). There was a slight trend for blue tits with lower AR to have a higher take-off performance (higher *P*_CoM_; *F*_1,27_=2.76, *r*^2^=0.09, *P*=0.11; Fig. 3C), even though there was a positive relationship between *P*_CoM_ and wing span (*F*_1,27_=4.53, *r*^2^=0.14, *P*<0.05). Individuals with lower AR did have significantly higher *P*_aero_ (*F*_1,27_=9.14, *r*^2^=0.25, *P*<0.01; Fig. 3C). The *P*_CoM_ was not related to the *M*_b_ of a bird (*F*_1,27_=0.14, *r*^2^=0.01, *P*=0.72).

**Fig. 4.**
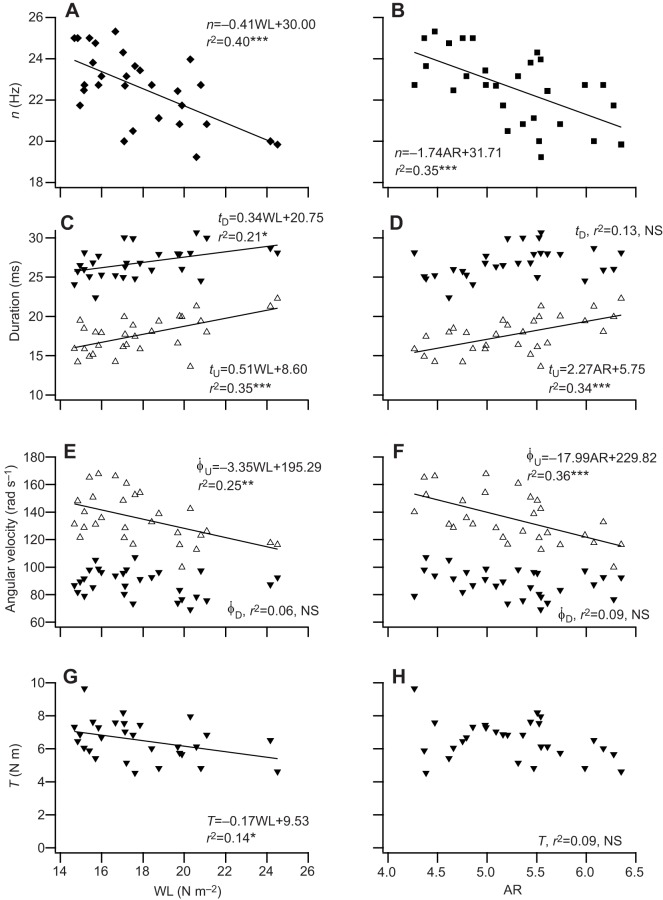
**Relationship between wing beat kinematics and wing morphology.** Least-squares regression of wing beat frequency (*n*) against (A) WL and (B) AR; downstroke duration (*t*_D_) and upstroke duration (*t*_U_) against (C) WL and (D) AR; angular velocity of the wing on the downstroke 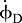 and on the upstroke 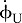 against (E) WL and (F) AR; and torque (*T*) against (G) WL and (H) AR. (*P<0.05, **P<0.01 and ***P<0.001, *N*=29.)

**Fig. 5.**
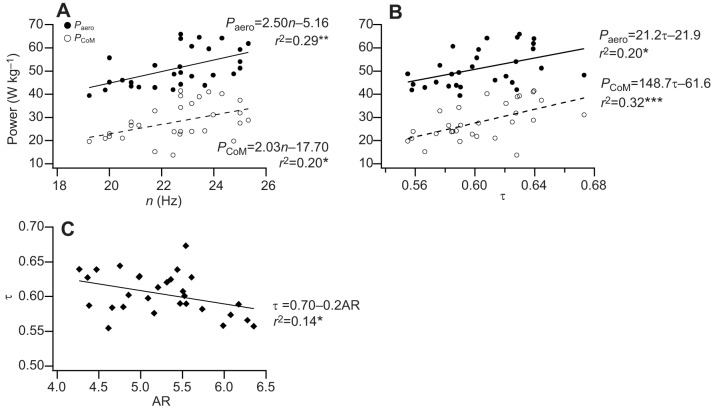
**The relationship between flight take-off performance and wing kinematics.** *P*_aero_ and take-off performance (*P*_CoM_) as a function of (A) *n* and (B) downstroke ratio (τ) during escape take-off flight in blue tits. (C) Relationship between τ and AR. The solid lines are least-squares regressions (*P<0.05, **P<0.01 and ***P<0.001, *N*=29).

Birds with high WL (*F*_1,27_=18.10, *r*^2^=0.40, *P*<0.001) and a high AR (*F*_1,27_=14.35, *r*^2^=0.35, *P*<0.001) had a lower wing beat frequency (*n*; Fig. 4A,B), after removing season and site from the model as neither improved the fit of the model to the data. Downstroke duration increased with increasing WL (*t*_D_, *F*_1, 27_=7.28, *r*^2^=0.21, *P*<0.05; Fig. 4C); upstroke duration increased with increasing WL (*t*_U_, *F*_1,27_=14.46, *r*^2^=0.35, *P*<0.001; Fig. 4C) and increasing AR (*t*_U_, *F*_1,27_=14.16, *r*^2^=0.34, *P*<0.001; Fig. 4D). The angular velocity of the wing during the downstroke was approximately constant and not related to either WL (*F*_1,27_=2.66, *r*^2^=0.09, *P*<0.11; Fig. 4E) or AR (*F*_1,27_=1.70, *r*^2^=0.06, *P*<0.20; Fig. 4F); the angular velocity of the wing on the upstroke decreased with increasing WL (*F*_1,27_=9.07, *r*^2^=0.25, *P*<0.01; Fig. 4E) and decreased with increasing AR (*F*_1,27_=14.95, *r*^2^=0.36, *P*<0.001; Fig. 4F). Torque (*T*) was inversely related to WL, decreasing with increasing WL (*T*, *F*_1,27_=4.49, *r*^2^=0.14, *P*<0.05; Fig. 4G), but was not significantly related to AR (*T*, *F*_1,27_=2.78, *r*^2^=0.09, *P*=0.11; Fig. 4H). Both *P*_aero_ (*F*_1,27_=10.89, *r*^2^=0.29, *P*<0.01) and *P*_CoM_ (*F*_1,27_=6.76, *r*^2^=0.20, *P*<0.05) increased with increasing *n* (Fig. 5A) and also with increasing τ (*P*_aero_, *F*_1,27_=6.78, *r*^2^=0.20, *P*<0.05; and *P*_CoM_, *F*_1,27_=12.61, *r*^2^=0.32, *P*<0.001; Fig. 5B). τ decreased with increasing AR (*F*_1,27_=4.51, *r*^2^=0.14, *P*<0.05; Fig. 5C). Models testing for the relationship between *P*_aero_ (*F*_2,26_=1.45, *P*=0.25), *P*_CoM_ (*F*_2,26_=0.45, *P*=0.64) and wing beat amplitude (Φ), and between *P*_aero_ (*F*_2,26_=1.92, *P*=0.17), *P*_CoM_ (*F*_2,26_=0.40, *P*=0.68) and stroke plane angle (β), that included season and site did not improve the models’ fit to the data.

## DISCUSSION

### Wing morphology and take-off performance

Take-off performance during escape flights (assessed as the sum of the rates of change of potential and kinetic energies of the centre of mass, *Ė*_P_ and *Ė*_K,ext_) decreased with increasing WL. The increased take-off performance of birds with a lower WL largely resulted from them having a higher acceleration of the CoM, and consequently a higher rate of change of kinetic energy compared with birds with a higher WL. While it was hypothesised that individuals with higher AR would have increased take-off performance, no significant relationship was found between *P*_CoM_ and AR. In the birds in this study, differences in WL and AR resulted from differences in wing area (rather than variation in *M*_b_ and wing span). As lift is proportional to wing area (Norberg, 1990), having a low WL may increase take-off performance because of the increased lift production in relation to body mass, enabling birds with a low WL to use a greater proportion of their flight muscle power output to increase the mechanical energy of the CoM.

Variation in wing area (and its concomitant effect on WL and AR) was related to seasonal moult. Blue tits began to moult after the breeding season (between May and June), with adults and juveniles undergoing their post-breeding and juvenile moults, respectively, beginning in June and ending in July. During moult, there were gaps in the wing, reducing wing area and increasing WL. Blue tits caught in June either had just started to moult or had not begun to moult. This may explain why the WL was higher in July, when both adults and juveniles would be in the midst of moulting, compared with June (pre-moult) and September (moult nearing completion). Primary feathers are moulted descendantly (away from the body towards the tip of the wing) whereas secondary feathers are moulted in the opposing direction; therefore, gaps in the wing due to feather loss are more likely to be mid-wing during July and at the wing tip in September. The higher AR in June and July compared with September was not the result of changes in wing span but was a consequence of the loss of wing area. Increases in AR via reductions in wing area are not expected to increase take-off performance. It is therefore changes in wing area that appear to have the greatest effect on take-off performance.

Previous studies have reported a similar decrease in take-off performance with increasing WL, also arising from reductions in wing area (during simulated or natural moult) and/or changes in body mass. For example, in response to simulated moult (in which wing area was reduced by removal of parts of feathers), take-off performance was found to be reduced, largely as a result of a reduction in flight velocity and in some cases an additional reduction in take-off angle (Swaddle et al., 1999; Lind, 2001). During natural moult, findings are more varied. In starlings, take-off performance was reduced as a result of the birds taking off at a shallower angle with no effect of take-off velocity (Swaddle and Witter, 1997; Williams and Swaddle, 2003). Increased WL resulting from increased body mass has been found to decrease take-off performance via a decrease in take-off angle (Kullberg et al., 2002; Lee et al., 1996), take-off velocity (Kullberg et al., 2002), or both take-off velocity and angle (Kullberg et al., 1996). However, in several studies, take-off performance was not adversely affected by changes in wing area or body mass. For example, there was no detrimental effect of reduced wing area during natural moult in tree sparrows (Lind, 2001) or during increased fat loading in willow tits and great tits (Kullberg, 1998; Kullberg et al., 1998), suggesting compensatory physiological adaptation for the changes in wing area or load. Increases in flight muscle mass with increasing body mass (Marsh, 1984), increases in relative flight muscle mass, or a reduction in body mass in response to reduced wing area during natural moult (Lind and Jakobsson, 2001; Swaddle and Witter, 1997) are all means by which birds may be able to maintain take-off performance despite changes in wing area or loading.

### Wing morphology and total take-off flight power requirements

In addition to calculating take-off performance (*P*_CoM_), an aerodynamic analysis was performed to calculate the total flight power requirements (*P*_aero_). It was hypothesised that *P*_aero_ would be constant between individuals, reflecting a constant power output and relative mass of the flight muscles. This was not found to be the case as *P*_aero_ was inversely related to WL. There are several potential reasons for this. First, it could indicate that birds with a higher WL have relatively smaller flight muscles, thereby producing less power per unit body mass. As all birds were released unharmed, it was not possible to establish whether there were differences in relative flight muscle mass, but they could be a potential determinant of take-off performance (Chai, 1997; Lind, 2001; Lind and Jakobsson, 2001). Second, a decrease in *P*_aero_ with increasing WL could result from birds with a higher WL operating with sub-maximal effort. We were unable to assess the level of motivation of individual birds. The manner in which flights were initiated was identical for all birds and so it seems likely that motivation was similar in all birds. Furthermore, individuals that did not take-off instantly were removed from the analysis. However, it cannot be ruled out that differences in WL could affect flight behaviour. Third, it could be due to birds with a higher WL having less powerful flight muscles, either by having weaker muscles per se or as a result of the muscles operating under suboptimal muscle length trajectories compared with birds with a lower WL. In grey catbirds, although there was hypertrophy of the pectoralis muscles in response to pre-migratory fat loading, there was no difference in muscle fibre type composition or the muscle's oxidative or glycolytic capacity (Marsh, 1984). However, the mechanical power output of the flight muscles could still vary despite the absence of changes in the physiological properties of the muscle. The mechanical power output of the flight muscles is determined by the pattern of motor unit recruitment, the physiological properties of the muscle and the length trajectory, which depends on the reciprocal interaction between the muscle properties and the load acting upon the wing as it moves through the air (Marsh, 1999; Askew and Marsh, 2002). Wings with differing WL and AR will experience a different aerodynamic force as they move through the air, thereby dictating the muscle's length trajectory and mechanical power output. Wing stroke kinematics were significantly related to wing morphology, with birds possessing a lower WL and a lower AR having a higher wing beat frequency, a relatively shorter downstroke and upstroke, but the same wing beat amplitude. These differences in kinematics result in the wings' downstroke velocity being approximately constant across all individuals. Therefore, the aerodynamic force generated by the wing is predicted to be higher in birds with a high wing area (low WL) compared with birds with a reduced wing area (high WL; the inverse relationship between torque and WL supports this prediction – see Fig. 4G). Increasing wing beat frequency and the proportion of the cycle spent shortening are both factors that can lead to an enhanced muscle mechanical power output during a cyclical contraction (Askew and Marsh, 1997) and could explain the increase in total aerodynamic power with decreasing WL and decreasing AR. Fourth, the aerodynamic model may not give reliable estimates of *P*_aero_, because of uncertainty in the appropriate values to use for the induced velocity correction factor *k* and *C*_D,pro_ (and whether these vary over the course of the wing stroke; Spedding and McArthur, 2010). The broad conclusions about variation in *P*_aero_ with WL would not be affected if any error was consistent within the species studied; however, this may not be the case and *k* and/or *C*_D,pro_ may vary with WL. Knowing the muscle mass and the muscle mass-specific power available for take-off would be an interesting area of further research as the power margin, the difference between the power available from the flight muscles and the aerodynamic power requirements (Norberg, 1990), could be compared.

### Concluding remarks

Birds maintain feather condition through seasonal moult. As a consequence of moult and its effects on wing area, blue tits showed seasonal variations in wing morphology (characterised by variation in WL and AR). The increase in WL resulting from moult incurs a cost in terms of a reduction in the escape take-off flight performance, which could increase the risk of predation. Decreased take-off ability and a potentially higher predation risk may be a cost of moult, but this is probably outweighed by the benefits of new flight feathers, and the subsequent increase in wing area and lift generation.
